# Optimization of Laminated Bio-Polymer Fabrication for Food Packaging Application: A Sustainable Plasma-Activated Approach

**DOI:** 10.3390/polym16131851

**Published:** 2024-06-28

**Authors:** Giacomo Foli, Filippo Capelli, Mariachiara Grande, Stefano Tagliabue, Matteo Gherardi, Matteo Minelli

**Affiliations:** 1 Interdepartmental Centre for Industrial Research–Advanced Mechanics and Materials (CIRI–MAM), University of Bologna–Viale del Risorgimento, 2, 40136 Bologna, Italy; filippo.capelli@unibo.it (F.C.); matteo.gherardi4@unibo.it (M.G.); matteo.minelli@unibo.it (M.M.); 2 Department of Civil, Chemical, Environmental, and Materials Engineering (DICAM), University of Bologna, Via Umberto Terracini, 28, 40131 Bologna, Italy; 3 Department of Industrial Engineering (DIN), University of Bologna, Via Umberto Terracini, 24, 40131 Bologna, Italy; mariachiara.grande2@unibo.it; 4 Corapack S.r.l., Via del Fontanile, 7, 22040 Brenna, Italy; s.tagliabue@corapack.com

**Keywords:** compostable packaging, plasma activation, polymers, composites, oxygen barrier

## Abstract

The current level of packaging consumption imposes a need to fabricate single-use food packaging with renewable and compostable materials, such as bio-polyesters (e.g., polylactic acid, PLA and polybutylene succinate, PBS) or cellulose, but their use is still problematic. Fabrication of bio-compostable composites can specifically address impeding challenges, and adhesive lamination, achieved with compostable glue, is becoming more and more popular with respect to the less versatile hot lamination. In this context, plasma activation, a chemical-free oxidation technique of a material’s surface, is used to increase the affinity of three different biomaterials (cellulose, PLA and PBS) toward a compostable polyurethane adhesive to decrease its amount by gluing bio-polyesters to cellulose. Optical Microscopy reveals activation conditions that do not affect the integrity of the materials, while Water Contact Analyses confirm the activation of the surfaces, with contact angles decreased to roughly 50 deg in all cases. Unexpectedly, ζ-potential analyses and subtractive infrared spectroscopy highlight how the activation performed superficially etches cellulose, while for both PLA and PBS, a general decrease in surface potential and an increase in superficial hydroxyl group populations confirm the achievement of the desired oxidation. Thus, we rationalize continuous activation conditions to treat PLA and PBS and to glue them to neat cellulose. While no beneficial effect is observed with activated PLA, bi-laminate composites fabricated with activated PBS fulfill the benchmark for adhesion strength using less than before, while oxygen permeation analyses exclude plasma-induced etching even at a nanoscale.

## 1. Introduction

Global production of polymeric materials has constantly grown since the early 1950s, reaching a yearly value of 460 million tons in 2019 [1], with roughly a third of that used for food packaging [2]. Single-use packaging represents the vast majority of this whole, but only less than 20% is collected for recycling, and inevitable impacts on waste management clearly demand tangible actions [3]. While lawmakers limit the utilization of single-use fossil-based materials for food packaging [4], researchers’ efforts are focused on the investigation and fabrication of new compostable bio-based polymeric materials [5,6,7,8,9]. At the present, the two most popular bio-compostable polymers obtained from renewable resources are polylactic acid (PLA) and polybutylene succinate (PBS). Briefly, PLA is polymerized from lactic acid, typically obtained from starch fermentation [10]. On the contrary, the two monomers required for the synthesis of PBS, 1,4-butanediol and succinic acid, can be prepared via bacterial fermentation of lignocellulosic sources or glycerol, the most abundant waste product of bio-diesel production [11,12].

Also, naturally occurring renewable polymers are currently being investigated, and cellulose is probably the most well-known representative [13,14,15]. Indeed, cellulose is a low-cost material that meets the majority of specific field requirements at first food contact [16,17], and its large worldwide availability guarantees a safe supply chain, allowing its usage as the food packaging material of the future [16,17]. However, some issues and limitations still exist and need to be overcome. For instance, a barrier to moisture and oxygen is a fundamental requisite [18,19,20], but it is well known that cellulose undergoes a huge drop in barrier performance at high humidity [21,22]. Such an issue has been addressed by coating the neat biomaterial with hydrophobic polymers [23]. The best solution currently available is a cellulose film coated with a thin layer made of Vinylidene Chloride (VdC) copolymers [24] that, given the extremely low amount of synthetic copolymer required, is still compostable [25]. Howevere, some issues are still present, and the heat-processing of the coated cellulose remains problematic. Indeed, the external hydrophobic layer allows hot sealing of the film [26], which is otherwise impossible with neat cellulose due to its thermal decomposition [27]. Nevertheless, VdC copolymers undergo thermal degradation at temperatures slightly above the melting point [28], consequently discouraging the use of hot lamination to fabricate multi-material laminates for specific food packaging markets, such as single-use coffee pods or ready-to-eat salads [26,27]. On the contrary, the roll-to-roll process for the fabrication of adhesive laminates is becoming a more and more attractive solution [29,30], but to preserve the compostability of the composite, the adhesives used must be compostable as well. To this aim, polyurethane adhesives are the best option, being both bio-degradable [31] and bio-based [32,33]. To make adhesive laminates competitive with the conventional heat-laminated solutions, the quantity of the applied adhesive must be minimized, and surface modification of the films to be glued can address such an issue [34,35]. Indeed, the presence of proper chemical groups on the material surface can promote interaction between adhesive precursors and the material itself. Dealing with polyurethane adhesive, hydroxyl groups play a crucial role [34], and an adequate oxidation process can increase their population on the surface of a material [36].

Oxidation of the surface of a material can be achieved by various approaches, as summarized below. Wet chemical processes, by means of strong oxidizing agents, are probably the most popular strategies followed in the past decades, but sustainability concerns strongly discourage such paths [37,38,39]. Also, flame treatments are currently avoided due to the need for hazardous or toxic chemicals [40,41,42]. On the contrary, plasma-activation treatments [43,44], which are performed at low [45,46] or atmospheric pressures [47], are gaining more and more attraction due to their relatively low carbon footprints [48,49]. The modification of material surfaces is originated by their interaction with a gaseous medium, which is locally ionized by a Corona [50] or a Dielectric Barrier Discharge, CD or DBD respectively [51,52]. Indeed, such an ionization results in the generation of highly reactive chemical species. By exposing the surface of a material (polymer, metal, silicon, etc.) to such a reactive environment, in-depth nanometric chemical modifications can be superficially achieved [53]. Most importantly, such a process is widely and easily tunable: modification of the electrical parameters of the discharge (mainly, tension and frequency) applied to the gaseous medium, together with treatment time, leads to significantly different results in terms of the final functionalization of material surfaces. Regarding process pressure, low-pressure plasma activations are preferred when 3D objects have to be functionalized [54]. In contrast, atmospheric pressure plasma activations are widely used to treat flat surfaces, and they are commonly employed in the fabric or polymeric film production industries [55,56]. In this last specific field, most employed device for the plasma activation is the previously mentioned DBD [51,52]. In these systems, two electrodes are spaced by a dielectric layer and in the free space between the electrodes, namely interelectrode gap (typically a few millimeters in thickness) plasma is locally formed. Atmospheric pressure plasma activation can be performed either in an inert or a reactive atmosphere. Working with inert gases, such as helium or argon, the discharge generates radicals on the surface of the material that, reacting with environmental oxygen or water molecules, produce polar functional groups on the material surface [57]. On the contrary, when a reactive medium such as air or oxygen is ionized, radical species are generated in the gaseous phase, and new functional groups are generated from reactions between such free radicals and the superficial chemical groups of the materials [58,59].

**Scheme 1:**
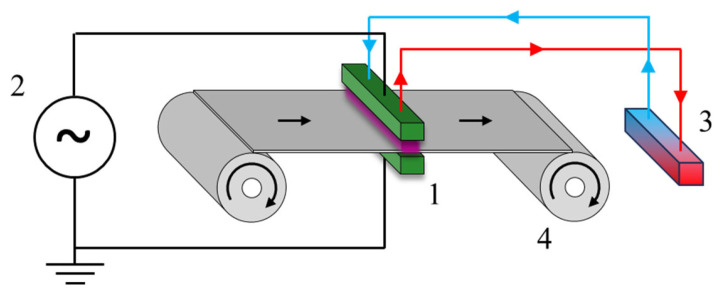
The plasma treatment system used was composed of 4 main elements: the DBD plasma source (1), the high-voltage generator (2), a cooling system (3) and the film handling system (4); further details are reported in Section 2.

In this work, three commercially available bio-polymeric films are plasma-activated at atmospheric pressure using air as the gaseous medium, to promote surface affinity towards a commercial and compostable polyurethane adhesive. Indeed, good results achieved dealing with glassy [60] or woody [61] materials prompted us to investigate the fabrication optimization of a new compostable composite material for food packaging. The reaction between plasma-generated species, mainly atomic oxygen and ozone [62], and superficial polymeric chains can introduce new chemical groups that are suitable to take part into the formation of polyurethane networks [63]. Most importantly, the use of plasma at atmospheric pressure is well known to limit the occurrence of plasma-induced material etching [53], which has a deleterious effect on oxygen barrier properties. Therefore, a VdC copolymer-coated cellulose (CLL) was coupled to PLA or PBS, to fabricate two different coupled composites: CLL//PLA and CLL//PBS. Surface modifications of polymeric films were initially carried out via a static process, and plasma-activated CLL was coupled to plasma-activated bio-polyester (PBS or PLA). We performed this to verify any possible increase in adhesion forces derived from the plasma activation and to investigate its origin. Later, we focused our attention on a continuous plasma activation to make the process scalable and industrially appealing, using a custom-made roll-to-roll machine, as presented in Scheme 1.

## 2. Materials and Methods

### 2.1. Materials

All the polymeric films and the compostable adhesive used in this work were kindly provided by Corapack S.r.l. (Brenna, Italy). The three polymeric films consisted of two different bio-polyesters, Poly Lactic Acid (PLA, EarthFirst, Ghent, Belgium) and Poly Butylene Succinate (PBS, IMB, Benevento, Italy), and a commercial cellulose coated with a thin layer of PolyVinylidene Chloride, PVdC (Nature Flex™, Futamura, Nagoya, Japan). The compostable adhesive was a polyurethane bicomponent glue (see Figure S1 in the Supplementary Materials) mixed in a ratio of 100/80 by weight. Potassium chloride (KCl), hydrochloric acid (HCl) and sodium hydroxide (NaOH) were purchased by Merck-Scientific and used as received. To prepare demineralized water, an OSMODEMI 4 Standard (Idrotecnica—Water Purification Systems, Genova, Italy) equipped with a reverse osmosis module and, following, a cartridge packed with mixed-bed ion-exchange resin, were used.

### 2.2. Procedure of Plasma Activation

Atmospheric plasma activation of the polymeric films was performed in air using a purpose-made dielectric barrier discharge (DBD). As shown in Figure S2, the DBD was made of 2 aluminum electrodes; the high-voltage electrode was liquid-cooled at room temperature to allow continuous operation, and it was covered with a two-component resin to avoid parasitic discharge. The dielectric layer, made of 3 mm ceramic gres, was placed on the bottom face of the high-voltage electrode. For this work, we used an interelectrode gap of 3 mm. The DBD was specifically designed to treat the full width of the films of interest; for this reason, the high-voltage electrode had a length of 26 cm and a total treatment area of 26 cm × 2.5 cm = 65 cm^2^.

For static treatments, the film was placed in the interelectrode gap and attached to the ground electrode with insulating tape to keep it taut and in place. For dynamic treatments, the tensioning and positioning of the film was managed by a roll-to-roll system. Once the sample to be treated was in place, the high-voltage generator was operated to trigger and sustain the plasma discharge for the predetermined treatment time. For dynamic treatments, this treatment time was determined by the forward speed of the film. The custom-made roll-to-roll machine for the continuous plasma activation of the films (Figure S3) was equipped with the DBD described before. The commercial high-voltage generator connected to the source (AlmaPULSE, AlmaPlasma srl, Bologna, Italy) was able to deliver up to 300 mA, with adjustable voltage (range: 0–20 kV) and frequency (range: 1–20 kHz).

### 2.3. Characterizations

#### 2.3.1. Optical Microscopy (OM) Analyses

All the Optical Microscopy images were acquired using a Zeiss Aixoskop 2 (Oberkochen, Germany) equipped with a Hall 100 illuminator. Digital images were captured with an AixoCam ICc 1 and elaborated using Carl Zeiss™ AxioVision Rel. 4.8.2 software.

#### 2.3.2. Water Contact Angle (WCA) Analyses

Water Contact Angles were determined using a drop shape analyzer (DSA30S-Kruss, Hamburg, Germany) depositing 4 μL of DI water drops.

#### 2.3.3. Adhesion Tests (T-Peel Test)

All adhesion tests were conducted according to the ASTM standard D-1876 Standard Test Method for Peel Resistance of Adhesive (T-peel test) [64]. Specifically, neat or plasma-activated films of the two bio-polymers were glued to the neat or plasma-activated films of commercial cellulose. Later, the glued composites were cut in stripes with a width of 2.5 cm and a length of 13 cm. Particular attention was paid to leaving the last 2 cm of the composite unbonded, i.e., free of adhesive, to prepare a T-shaped sample like the one presented in Figure S4.

An INSTRON^®^ 5966 (INSTRON^®^, Norwood, MA, USA), equipped with a 10 kN load cell, was used as the tension testing machine. The bent and unbounded ends of the tested samples were clamped to screw action grips (INSTRON^®^ 2710-102) spaced at a distance of 2 cm. The load was then applied at a constant speed of 10 mm·min^−1^ until the tested composite was completely separated. Data were collected with INSTON Bluehill 3 software. For each composite, at least 6 samples were prepared and tested; all the loads presented in this work, expressed in Newtons (N), are the average value ± standard deviation of the average load determined for each sample.

#### 2.3.4. Determination of the Surface Zeta-Potential (ζ-Potential)

All the ζ-potentials presented in this work were determined using an electrokinetic analyzer for solid surfaces (SurPASS 3, Antoon Paar, Graz, Austria). A 1 mM KCl water solution was used as the probing electrolyte, and 0.5 M solutions of HCl and NaOH were used to adjust the pH value of the electrolyte. Before each analysis, each sample was repeatedly rinsed (at least 5 times) with the electrolyte to stabilize the surface of the tested material at the pH of analysis. The ζ-potential measured at the end of the analyses was the average of 5 measurements conducted on the same sample. The presented values of ζ-potential are the average value ± standard deviation of 3 independently analyzed samples. When a plasma-activated material was analyzed, the ζ-potential was measured immediately after the plasma activation, and a new plasma-activated sample was loaded for every tested pH to avoid hydrophobic recovery issues.

#### 2.3.5. Attenuated Total Reflectance–Fourier Transform Infrared (ATR-FTIR) Characterizations

All the ATR-FTIR spectra were acquired using a Nicolet S20 (Thermo Scientific, Waltham, MA, USA) equipped with a germanium (Ge) crystal plate (ID7 ATR Ge, Thermo Scientific, USA). For each spectrum, 128 scans were acquired, with a resolution of 4 cm^−1^, in the range of 600–4000 cm^−1^. Prior to each acquisition, a background of 32 scans was acquired. The subtracted spectra presented in this work were obtained by processing the acquired spectra with OMNIC software (driver version: 9.11.745).

#### 2.3.6. Gas Transport Analysis

All gas transport analyses were performed at 23 °C according to a manometric technique: ASTM D-1434 [65]. All tested samples had an area of 2.2 cm^2^ and the applied Δp was in the range of 1.8–1.9 bars, except for CLL where, due to its extremely low permeability, a Δp of 4 bars was used. The increase in moles, n, of penetrant in the down-stream volume was determined by monitoring the increase in the down-stream pressure and applying the ideal gas equation of state:

n = (p · V)/(R · T)(1)

where p is the down-stream pressure, tracked in a timely manner and expressed in [Pa]; V is the value of the down-stream volume (V = 17.5 cm^3^ = 17.5 × 10^−6^ m^3^); R is the universal gas constant (R = 8.3145 Pa·m^3^·mol^−1^·K^−1^) and T is the temperature of the analysis (T = 23 °C = 296.15 K). The output of a typical permeation experiment is presented in Figure S5.

The permeability of the penetrant (P), expressed in (mol·m·s^−1^·m^−2^·Pa^−1^), was finally calculated from the steady-state slope, i.e., the steady-state flux [J = (d · mol)/(d · t)], according to Equation (2):

P = J′ · l/∆p(2)

where J′ is the steady-state flux J per unit of area, ∆p is the difference in pressure between the up- and the down-stream volumes and l is the thickness of the tested sample.

## 3. Results and Discussion

**Figure 1:**
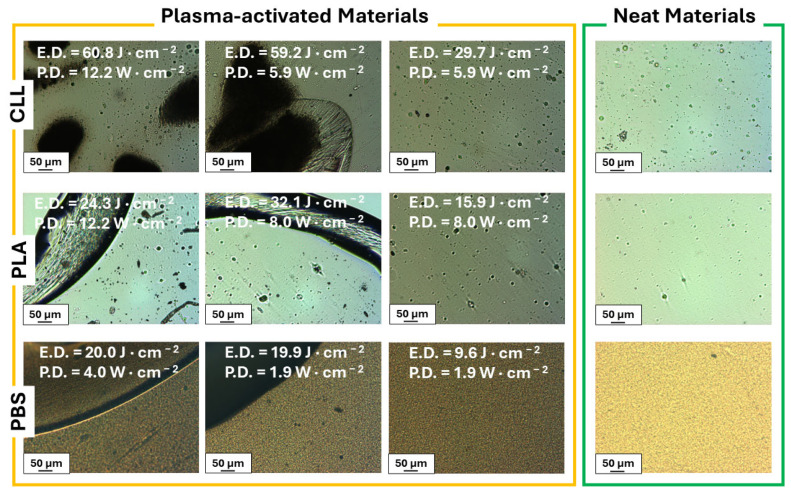
OM screening of the effects of diverse activation conditions on our three materials (plasma-activated materials, yellow box): only the conditions that did not attack integrity of the materials (third column from the left) were further investigated; for sake of comparison, OM images of three neat materials are reported (neat materials, green box).

The intensity of plasma activation can be tuned by varying the voltage and frequency of the electrical discharge [66], as well as changing the treatment time. In case of a static process, the latter one will be equal to the exposure time of the film to ionized atmosphere. On the contrary, in a continuous process, it is calculated as the residence time of the film in the plasma-irradiated area, which depends on the operating speed of the roll-to-roll machine used. Despite such considerations, the search for the highest intensity can lead to etching of the material, even if we opt for less aggressive atmospheric-pressure plasma activation. Thus, we initially screen a wide range of operative conditions of the plasma to exclude the ones that clearly attack the surface integrity of our materials. We analyze the integrity of the differently treated materials by Optical Microscopy (OM) (see Figure 1), focusing our attention only on the operative conditions that do not microscopically deteriorate the films. Then we verify the effectiveness of the activation by recording the Water Contact Angle (WCA). Indeed, the superficial insertion of oxygen-containing functionalities alter the wettability of the polymers, lowering the WCA. The best results in terms of WCA, and the details about the usedplasma-condition combinations, are reported in Table 1.

**Figure 2:**
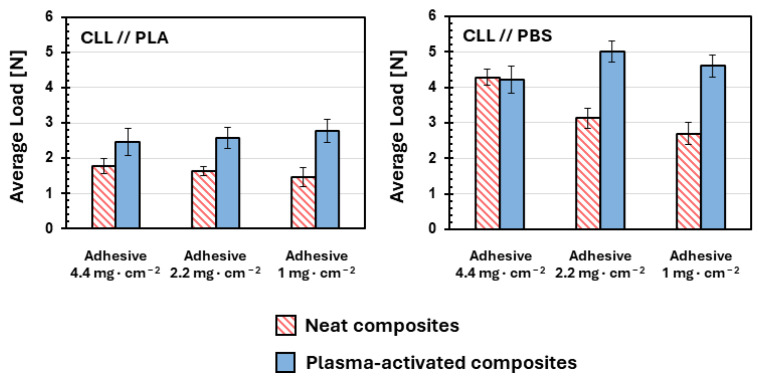
Average loads recorded during the T-peel tests conducted on the various coupled bi-laminates prepared in diverse coupling conditions: cellulose and PLA on the left; cellulose and PBS on the right; for all coupled bilayers, at least 5 specimens were prepared and tested; error bars represent corresponding standard deviations.

Comforted by promising results, we proceed with the fabrication of composites using a reference amount of commercial adhesive: 4.4 mg·cm^−2^. We choose such a value considering the quantity of polyurethane adhesive that is usually spread in similar works [67]. In detail, we spread adhesive on the surface of the films immediately after the activations, to avoid significant aging phenomena due to well-known hydrophobic recovery phenomenon [68,69]. Simultaneously, control composites prepared with no plasma-activated films, are prepared. The results of the adhesion tests and T-peel tests [64] are reported in Figure 2.

In the case of the CLL//PLA composites (Figure 2, left), the effect of plasma activations is immediately evident. The adhesion forces of activated composites are enhanced with respect to the poor value recorded for the untreated composite, with 2.5 N vs. 1.8 N, respectively. Conversely, analyses of the plasma-activated CLL//PBS composites show no significant increase in adhesion force after the activation (Figure 2, right). However, looking at the absolute values of strength, it is evident the adhesive used interacts significantly better with PBS with respect to PLA, and the adhesion force of untreated PLA is almost 60% lower with respect to untreated PBS, with 1.8 N vs. 4.2 N. Such an observation is in agreement with the pre-plasma WCAs reported in Table 1: the more hydrophilic PBS (WCA = 63.4 deg) reasonably interacts better with the polyurethane adhesive compared to the more hydrophobic PLA (WCA = 79.7 deg). For this reason, we decide to investigate the effect of plasma activation, lowering the amount of applied adhesive. We prepare same composites but using half (2.2 mg·cm^−2^) and less than a quarter (1 mg·cm^−2^) of the adhesive. Favorably, in all activated composites, no significant decreases in adhesive strengths are observed and comparable values of the loads are observed when lowering the amount of applied adhesive down to 1 mg·cm^−2^, independently of the bio-polyester used. On the contrary, a linear decrease in adhesion force for non-plasma-activated composites is evident, as shown in Figure 2.

**Figure 3:**
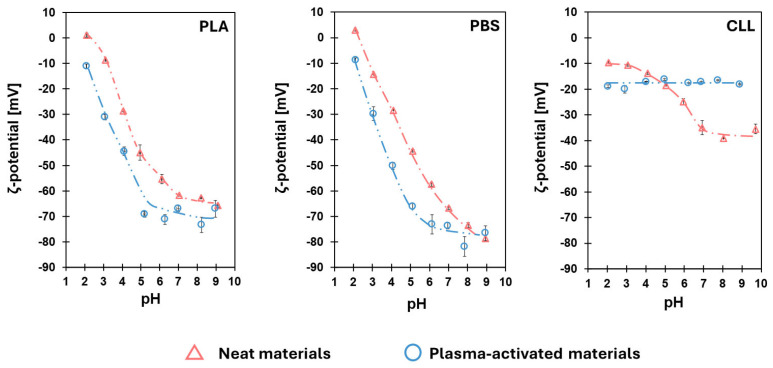
ζ-potential–pH curves of the surfaces of the three studied materials recorded before (neat materials: red triangle, dashed and pointed lines) and immediately after the activation treatments (plasma-activated materials: blue circle, dashed and double-pointed lines).

To better inspect the effect of the plasma activation on the film surfaces, we record the ζ-potential–pH curves, a technique widely used to investigate differences obtained after oxidation of materials surfaces [70]. The curves obtained for the neat films (Figure 3: red triangles, dashed and pointed lines) are in line with what is expected, given the chemical nature of the materials. Indeed, our bio-polyesters present pH-sensitive groups on the surface, mainly carboxylic and hydroxyl functionalities, and quite steeped ζ-potential–pH curves are recorded. Indeed, at a low pH, all carboxylic moieties are protonated, and the slightly positive ζ-potential values observed must be ascribed to the partial protonation of the hydroxyl groups, which are characterized by a protic character [71]. Increasing the pH to basic conditions, no protonation of hydroxyl groups is possible anymore, and all carboxylic groups are deprotonated; consequently, negative ζ-potentials are recorded. In the case of neat CCL, however, the ζ-potential curve still depends on the pH, but this curve is less pronounced (Figure 3, right: red triangles, dashed and pointed lines). The hydrophobic coating present on the CLL film, indeed, is scarcely polarizable.

After the plasma activations and consequent insertions of oxygen-containing groups, most of the surface properties are affected, including the resulting ζ-potential [72]. The curves recorded for PLA and PBS indicate that plasma activation has modified the superficial charges (Figure 3, left and central: blue circles, dashed and double-pointed lines). In detail, for both plasma-activated bio-polyesters, more negative ζ-potentials are observed, consistently with an augmented population of protonable groups on the surface of the bio-polyesters. Indeed, reactive species, generated in the ionized atmosphere, react with polymeric surface, leading to the scission of polyester chains [73]. The final result is the formation of new carboxylic and protonable hydroxyl groups that alter the response of the surface of the materials to pH modulation. Conversely, a pH-independent ζ-potential curve, fluctuating around −18 mV, is recorded for the plasma-activated CLL (Figure 3, right: blue circles, dashed and double-pointed lines). This peculiar behavior of the superficial charge is significantly different from the activated bio-polyesters and from the curve recorded for the neat CLL. Moreover, the ζ-potential results are in contrast with the conclusion derived from the WCA analyses (see Table 1). While a decrease in the WCA of the plasma-activated CLL points out an increase in wettability, the ζ-potential–pH curve does not reveal any footprint of the presence of protonable hydroxyls species.

**Figure 4:**
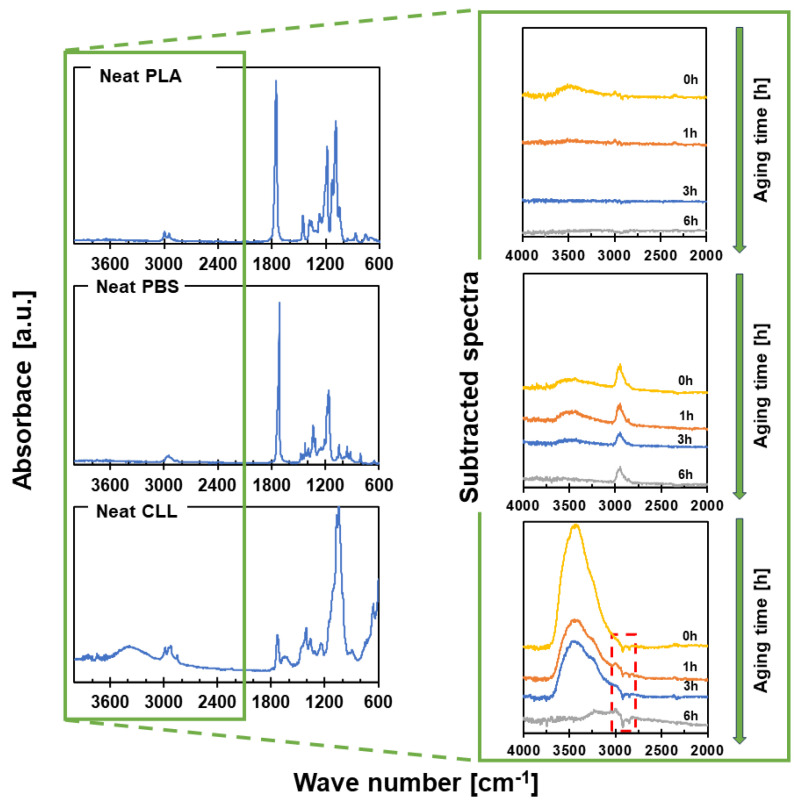
On the left, ATR-FTIR spectra of the neat materials are presented: PLA, PBS and CLL (commercial cellulose); on the right, subtracted ATR-FTIR spectra with respect to corresponding neat spectra, recorded at various times after plasma activation, are presented; for detailed assignations of all the peaks, see Table S1 in the Supplementary Materials.

The anomaly of the ζ-potential–pH curve of the activated CLL is inspected by ATR-FTIR analyses of the three materials, recording the spectra before and after plasma activation and paying particular attention to the spectral region of the hydroxyl groups, 3000–4000 cm^−1^ [74]. Moreover, to investigate the entity of hydrophobic recovery, we also acquire spectra of activated materials at various times after plasma activation. All results are reported in Figure 4.

The subtracted ATR-FTIR spectra (Figure 4, on the right) reveal that, in the case of PLA, plasma activation slightly increases the concentration of superficial hydroxyl moieties (Figure 4 on the right, top subtracted spectrum, peak at 3400 cm^−1^). Moreover, this activation is also quite instable, to the point that, after only 1 h after the activation, no traces of additional hydroxyl groups are visible. On the contrary, a more pronounced and stable insertion of hydroxyl functionalities is achieved on PBS (Figure 4 on the right, central subtracted spectrum), with the usual broad and well-visible peak at 3400 cm^−1^ that lasts up to 3 h after the activation. Nevertheless, the most pronounced plasma activation is observed for commercial cellulose CLL (Figure 4 on the right, bottom subtracted spectrum). A huge increase in superficial hydroxyl groups is evident immediately after plasma activation, and it is still well visible after 3 h. Intriguingly, the results obtained for CLL keep puzzling any reasoning, and only a deeper examination of the subtracted spectra finally allows us to understand real effect of the plasma activations., As highlighted by the red-dashed box in Figure 4 (bottom, right corner), some negative peaks are visible in all the CLL subtracted spectra, at roughly 2900–3000 cm^−1^. The presence of negative peaks in our CLL subtracted spectra means that plasma activation removes chemical groups from the surface, instead inducing functionalization. Specifically, signals of removed chemical groups are in the region of the characteristic VdC peaks [75], which are the main component of the hydrophobic coating present on our CLL. This observation finally allows us to pick up the pieces of our investigation. While for both bio-polyesters, plasma activation results in the awaited functionalization induced by reactive species generated into ionized atmosphere, something unexpected takes place when dealing with CLL. Indeed, the performed plasma activation of CLL provokes surface etching of the film, and the thin superficial hydrophobic layer is removed. This conclusion agrees with both the WCA and the ζ-potential analyses. As a matter of fact, the removal of the hydrophobic coating exposes bare cellulose to the surface, and a lower WCA is recorded. Moreover, the peculiar ζ-potential–pH curve recorded for plasma-activated CLL matches the one acquired for pure cellulose [76]. Considering the deleterious effect that the removal of such a coating could have on gas barrier performance, we decide to determine the gas permeability of CLL to O_2_ before and after plasma activation (see Section 2.3.6 for details). As expected, a drop in the O_2_ barrier takes place after plasma activation: the extremely low permeability to O_2_ recorded for neat CLL (2.6 × 10^−20^ mol·m·m^−2^·s^−1^·Pa^−1^) rises after the plasma activation (32.8 × 10^−20^ mol·m·m^−2^·s^−1^·Pa^−1^). For this reason, we realize that plasma activation of the commercial cellulose used is not convenient. The beneficial effects observed in terms of adhesion enhancement are accompanied by an undesired etching of the hydrophobic coating, which is fundamental to achieve superior gas barrier performance.

After the conclusion of this preliminary study, we proceed to the development of a continuous activation process. Since the CLL is etched by a plasma-ionized atmosphere, with negative effects on the gas barrier performance of the film, a continuous plasma activation is only developed for our two bio-polyesters. Moreover, because we observe good adhesion values even when lowering the amount of glue applied (see Figure 2), both composites are prepared using only 1 mg·cm^−2^ of adhesive. First, we rationalize new operative activation conditions for PLA and PBS to be used on a custom-made roll-to-roll machine developed for plasma activation. Dealing with a continuous process, the time of exposition to a plasma-ionized atmosphere is related to the operational speed of the roll-to-roll machine and to the size of the plasma-irradiated region. For this preliminary work, we define a plasma-irradiation length of 2.5 cm, and we opt for an operating speed of 14.5 cm·s^−1^, a common choice when dealing with such investigations [77]. We then rationalize the continuous plasma activation conditions, starting from the above-presented static conditions. Indeed, our continuous process dramatically reduces the exposition time of the materials to the plasma source. While some seconds are necessary to statically plasma activate PLA and PBS, both the dimension of our plasma source (2.5 cm) and the chosen operating speed (14.5 cm·s^−1^) reduce these values to less than 0.2 s. For this reason, we identify new activation conditions that permit us to achieve a comparable power and energy density per unit of time, searching for a retention of these two parameters from the static to the continuous process. The continuous plasma activation conditions for the two bio-polyesters are reported in Table 2.

**Figure 5:**
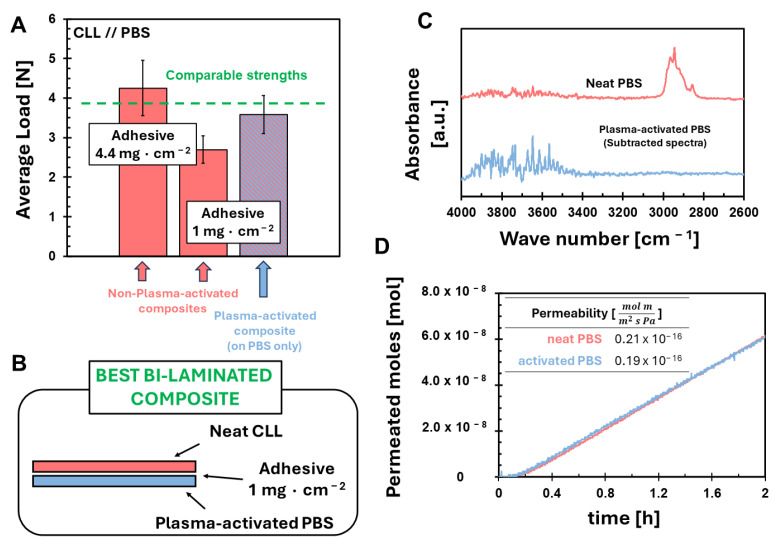
Characterization of continuously plasma-activated PBS; (**A**) comparison of adhesion strengths of the prepared composites expressed in terms of average load [N]; (**B**) schematic representation of the fabrication procedure of the best bi-laminated composite; (**C**) FTIR spectrum of neat PBS (blue line) and the corresponding subtracted spectrum after PBS continuous plasma activation (orange line) and (**D**) oxygen permeation determined at 23 °C using a permeation area of 2.2 cm^2^ and ΔP = 1.9 bars.

Working as before, we prepare the two composites, and we perform adhesion tests, following the usual standard procedure [64]. In Figure S6, we report the typical outputs of the adhesion tests for these last two composites. The almost constant value of the loads recorded during the two different analyses testifies to the goodness of the achieved adhesion. For the PLA-based composite, we find out that our best continuous plasma activation of the bio-polyesters is not sufficient to achieve any effect on adhesion strength. Indeed, no increase in adhesion is observed with respect to the corresponding non-plasma-activated composite (Figure S7). These results cannot be considered unexpected, since, even with the static plasma activation, a poor hydroxyl functionalization is achieved for PLA (Figure 4). Thus, we conclude that the good results observed in Figure 2 are mainly due to the plasma etching of the hydrophobic coating of CLL and the consequent exposure of the cellulose hydroxyl groups to the adhesive precursors. On the contrary, appreciable results in terms of adhesion are observed for the composite prepared with the continuously plasma-activated PBS. Indeed, PBS-based composite, fabricated with only 1 mg·cm^−2^, turns out to have an adhesion strength that is 30% higher than the corresponding non-plasma-activated composite. Moreover, the value of load is comparable to the strengths observed for the corresponding non-plasma-activated composite but prepared with 4.4 mg·cm^−2^; see Figure 5A. A schematic representation of the fabrication procedure of this composite is represented in Figure 5B. Even in this case, the formation of hydroxyl groups is confirmed by subtractive ATR-FTIR spectroscopy, as shown in Figure 5C. Finally, we verify the absence of etching by determining the permeability of PBS to O_2_ before and after the plasma activation (Figure 5D). The comparable values of permeabilities exclude any damage of plasma-activated PBS even at a nanoscale.

Finally, we are able to prepare a fully bio-degradable adhesive laminate using a lower quantity of spread glue but maintaining the same laminate adhesion strengths. These results are obtained after a laborious optimization of the plasma-activation conditions that make the surface of our target material more prone to interact with the adhesive precursors. Most of all, our plasma activation does not provoke any damage to the treated material, leaving the gas barrier performance unchanged.

## 4. Conclusions

Taking advantage of plasma technology, we optimize the adhesive lamination of a fully compostable bi-laminate with potential applications in food packaging. We perform this to combine the properties of different bio-polymeric films by adhesive laminating a commercial cellulose film (CLL) to two different polyesters: PLA or PBS. The present preliminary studies reveal that plasma activation positively affects the propensity of all the three of the tested materials to adhesion, but, in the case of CLL, etching of the film takes place. Thus, we prepare the bi-material composites, performing plasma activation only on the two bio-polyesters. Sadly, when plasma-activated PLA is glued to neat CLL, no increase in adhesion performance is observed. On the contrary, an increase in adhesive strength is observed using plasma-activated PBS. Thus, we decrease the quantity of the loaded adhesive to further investigate the effectiveness of our plasma activation. At the end, we fabricate a bi-laminate composite made of plasma-activated PBS and neat CLL, attaining an adhesion strength comparable to the neat bi-laminate but using less than 25 wt% of adhesive.

## Figures and Tables

**Table 1:** Static plasma-condition combinations identified for the three studied materials and variation in WCAs.

Material	Voltage [kV]	Frequency [kHz]	Time [s]	Energy Density [J · cm^−2^]	Power Density [W · cm^−2^]	WCA [deg]	
						Pre- Plasma	Post- Plasma
CLL	12	20	5	29.7	5.9	85.9 ± 2.8	54.2 ± 4.8
PLA	14	20	2	15.9	8.0	79.7 ± 1.6	47.4 ± 3.6
PBS	14	5	5	9.6	1.9	63.4 ± 3.8	49.9 ± 1.3

**Table 2:** Continuous plasma operating conditions identified for the PLA and PBS films.

Material	Tension [kV]	Frequency [kHz]	Energy Density [J · cm^−2^]	Power Density [W · cm^−2^]
PLA	10	2	27.2	152.3
PBS	12	10	9.9	55

## Data Availability

The original contributions presented in the study are included in the article and in the Supplementary Materials; further inquiries can be directed to the corresponding author.

## Supplementary Materials

The following Supplementary Materials can be downloaded at https://www.mdpi.com/article/10.3390/polym16131851/s1: Figure S1. Schematic representation of the samples prepared for T-peel tests; Figure S2. Output of a typical permeation experiment: the collected experimental data (black points) are elaborated to determine the steady-state slope (red line) used for the calculation of permeability values; Figure S3. Sources used for plasma activation. 1—high-voltage electrode, 2—ground electrode, 3—two-component resin, 4—high-voltage connection, 5—cooling connection, 6—plasma discharge; Figure S4. Roll-to-roll machine used for the continuous plasma activation; Figure S5. ATR-FTIR of the adhesive mixture at zero cross-linking time (blue line) and after 24 h at 35 °C (grey line); Figure S6. Output of a typical adhesion test: the composites were fabricated using neat CLL and the continuously plasma-activated PLA (on the left) of continuously plasma-activated PBS (on the right), using 1 mg·cm^−2^ of adhesive in both cases; Figure S7. Adhesion strengths determined for the bi-laminates fabricated with continuously plasma-activated PLA or PBS coupled with neat commercial cellulose, using 1 mg·cm^−2^ of adhesive. Table S1: Assignation of the peaks reported in the three spectra reported in Figure 4. References [78,79,80,81] are cited in the Supplementary Materials.
