# Supplementary material for: Optimization of Laminated Bio-Polymer Fabrication for Food Packaging Application: A Sustainable Plasma-Activated Approach
Source: Polymers (Basel). 2024 Jun 28;16(13):1851. doi: 10.3390/polym16131851 (PMC11244328; doi:10.3390/polym16131851)
Supplement: Supplementary file 1 [file polymers-16-01851-s001.zip › polymers-3034593-supplementary.pdf]

Optimization of laminated bio-polymers fabrication for food-packaging application: a sustainable plasma-activated approach

Giacomo Foli, Filippo Capelli, Mariachiara Grande, Stefano Tagliabue, Matteo Gherardi, Matteo Minelli

1. Compostable adhesive: study of the cross-linking process

The chemistry of the compostable commercial adhesive used in this work. According to the guidelines of the supplier, cross-linking of the adhesive is accomplished in less the 24 hours thought a promoted process performed at room temperature. Thus, we compared the ATR-FTIR spectrum of the adhesive recorded at time zero after mixing and after 24 hours after mixing, see **Figure S5**.

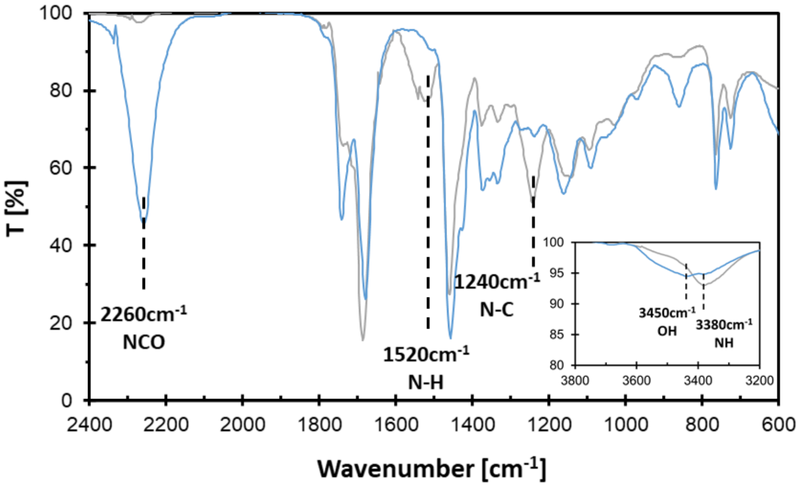

Figure S1. ATR-FTIR of the adhesive mixture at zero cross-linking time (blue line) and after 24 hours at 35 °C (grey line).

One can readily observed that the pronounced band at roughly 2260 cm^{-1} present in the adhesive at time zero, and ascribable to isocyanate [78], almost disappear after 24h. Moreover, appearance of peaks at 1520 cm^{-1} and 1240 cm^{-1} 24h after the mixing further support cross-linking mechanism. Indeed, these peaks correspond to N-H bending and N-C stretching respectively, functionalities both present in the urethane linkage. Finally, the broad signal at 3450 cm^{-1} well visible before the cross-linking, and ascribable to hydroxyl groups, completely disappear after the cure, while a new peak at 3380 cm^{-1} appeared: N-H stretching [79,80]. Our preliminary study confirmed the polyurethane nature of the used adhesive.

2. Procedure of the plasma-activation

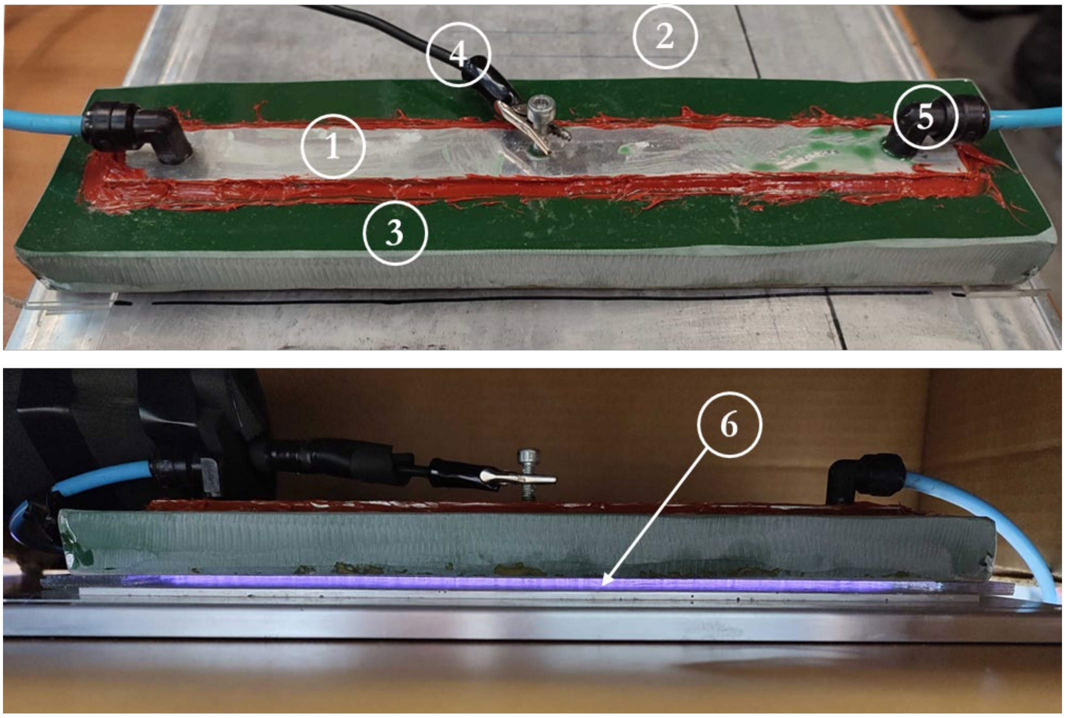

Figure S2. Source used for the plasma activation. 1 - high voltage electrode, 2 – ground electrode, 3 – two-components resin, 4 – high voltage connection, 5 – cooling connection, 6 – plasma discharge.

2.1 Roll-to-roll machine

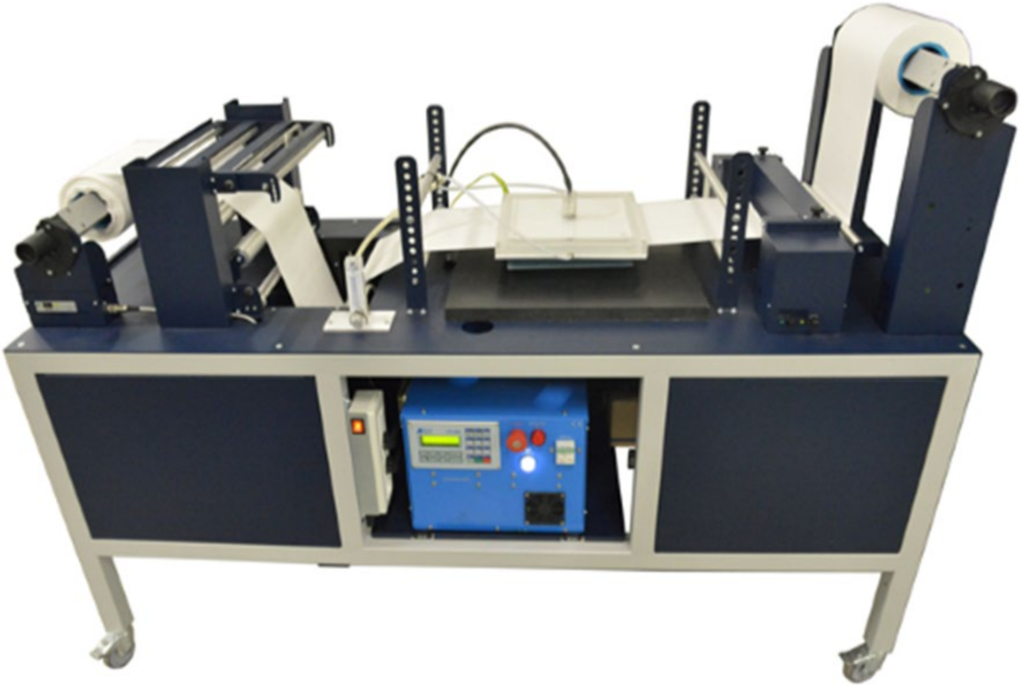

Figure S3. Roll-to-roll machine used for the continuous plasma activation.

3. Adhesion Tests (T-Peel Test)

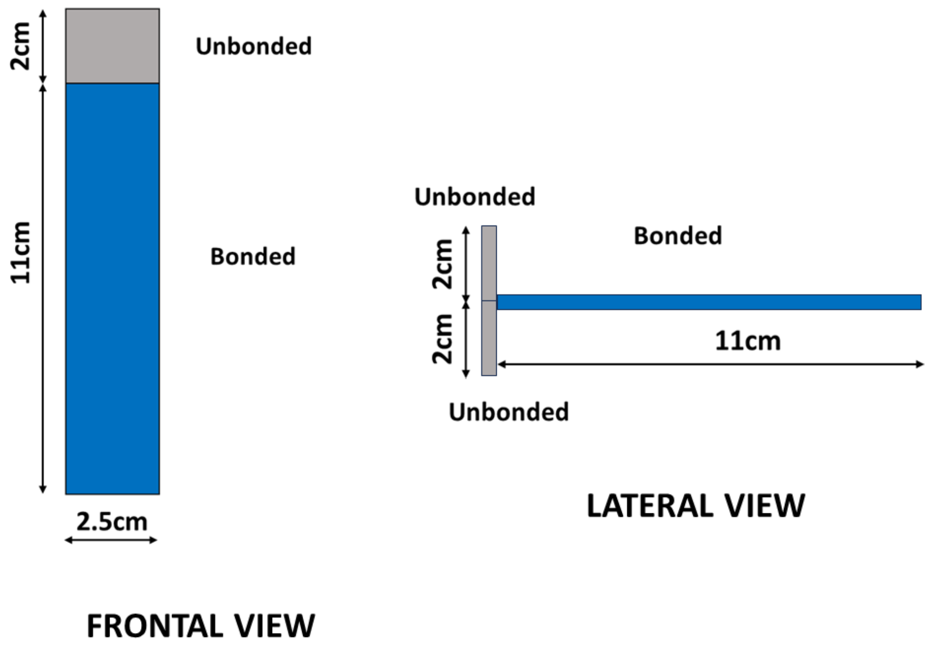

Figure S4. Schematic representation of the samples prepared for T-Peel Tests: on the left, the frontal view of a typic al sample; on the right, the lateral view, where the T-shape of the sample is well visible.

4. Gas transport analysis

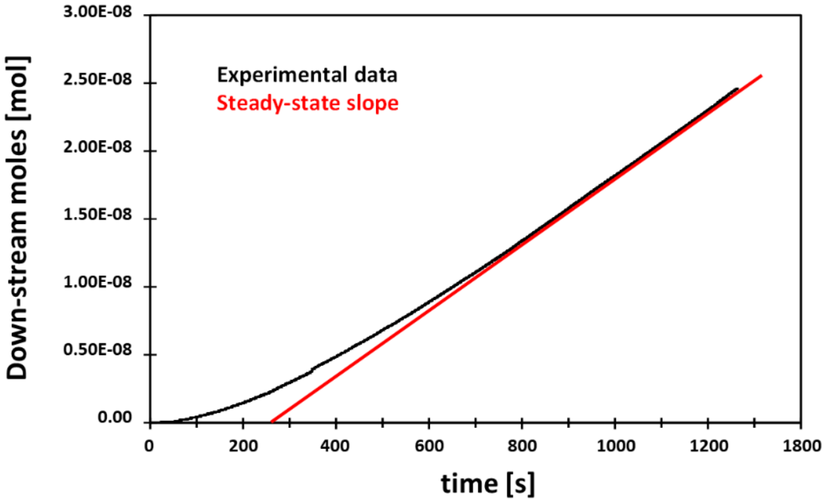

Figure S5. Output of a typical permeation experiment: the collected experimental data (black points) are elaborated to determine the steady-state slope (red line), used for the calculation of permeability values.

5. ATR-FTIR spectra: assignation of the peaks

Table S1. Assignation of the peaks reported in the three spectra reported in **Figure 4**.

Material	Peaks	Chemical groups
CLL [81,82]	3600 - 3100 cm^{-1}	OH, stretching
	3000 - 2900 cm^{-1}	CH_2, stretching (of the bulk cellulose)
		CH_2, stretching (of the hydrophobic coating, PVdC)
	1650 cm^{-1}	OH, bending
	1500 - 1400 cm^{-1}	CH_2, bending
	1200-1000 cm^{-1}	C-O, stretching
PLA [83]	3000 - 2900 cm^{-1}	CH_2, stretching
	≈ 1750 cm^{-1}	C=O
	1500 - 1400 cm^{-1}	CH_2, bending
	1200 - 1000 cm^{-1}	(C-O), stretching
PBS [6]	3000 - 2900 cm^{-1}	CH_2, stretching
	≈ 1750 cm^{-1}	C=O
	1500 - 1400 cm^{-1}	CH_2, bending
	1200 - 1000 cm^{-1}	C-O, stretching

6. Continuous plasma-activation of bio-polymers: comparison of adhesion strengths

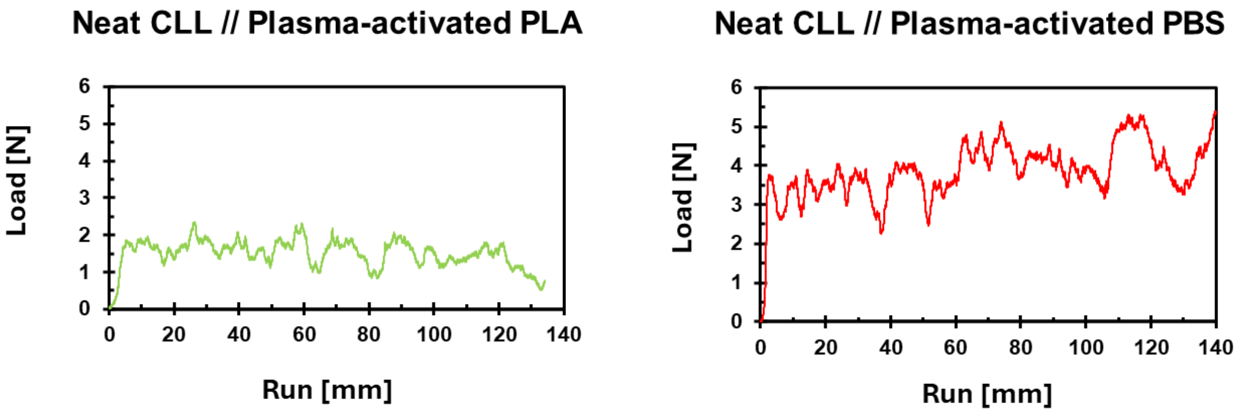

Figure S6. Output of a typical adhesion test: the composites were fabricated using neat CLL and continuously plasma-activated PLA (on the left) of continuously plasma-activated PBS (on the right), using in both cases 1 mg·cm^{-2} of adhesive.

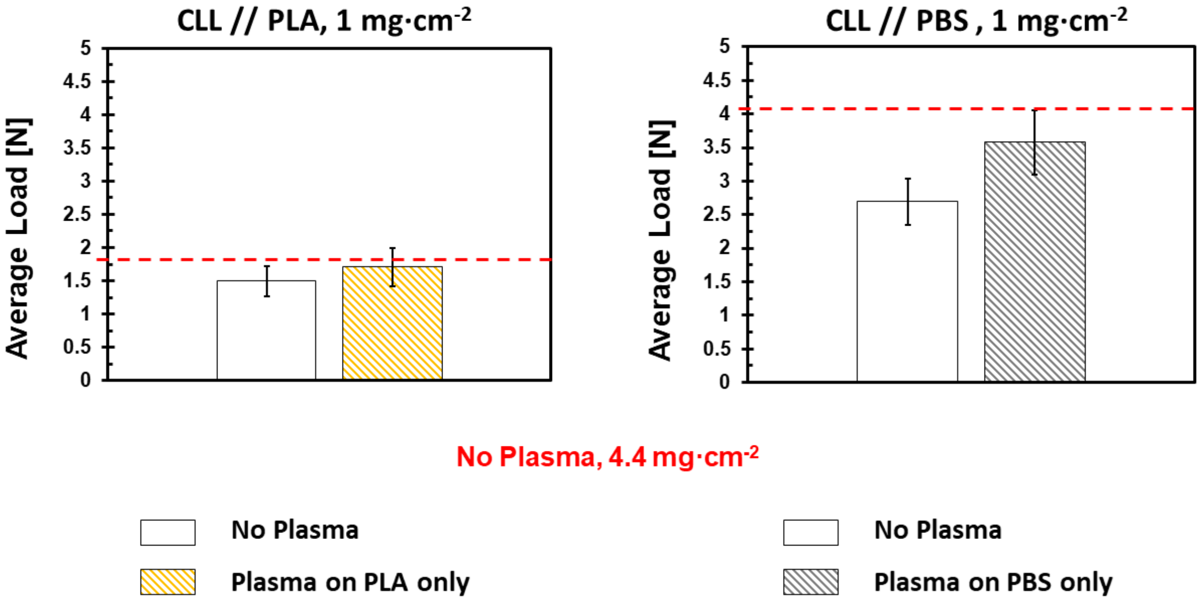

Figure S7. Adhesion strengths determined for the bi-laminates fabricated with continuous plasma-activated PLA or PBS coupled with the neat commercial cellulose, using 1 mg·cm^{-2} of adhesive.

References

78. Foli, G.; Degli Esposti, M.; Morselli, D.; Fabbri, P. Two-Step Solvent-Free Synthesis of Poly(Hydroxybutyrate)-Based Photocurable Resin with Potential Application in Stereolithography. *Macromol Rapid Commun* **2020**, *41, 1900660*, doi:10.1002/marc.201900660.

79. Choi, S.W.; Seo, D.W.; Lim, Y.D.; Jeong, Y.G.; Islam Mollah, M.S.; Park, H.; Hong, T.W.; Kim, W.G. Synthesis and Properties of Multihydroxy Soybean Oil from Soybean Oil and Polymeric Methylene-Diphenyl- 4,4′-Diisocyanate/Multihydroxy Soybean Oil Polyurethane Adhesive to Wood. *J Appl Polym Sci* **2011**, *121*, 764–769, doi:10.1002/app.33405.

80. Hooper, A.E.; Tompkins, H.G. Convenient Calibration of FTIR Peak “size” for Thin Organic/Polymer Films. *Surface and Interface Analysis* **2001**, *31*, 805–808, doi:10.1002/sia.1119.

81. Wei, X.; Lu, Q.; Sui, X.; Wang, Z.; Zhang, Y. Characterization of the Water-Insoluble Pyrolytic Cellulose from Cellulose Pyrolysis Oil. *J Anal Appl Pyrolysis* **2012**, *97*, 49–54.

82. Cataldo, F.; Ursini, O.; Ragni, P.; Lilla, V.; Angelini, G. Radiation-Induced Polymerization of Vinylidene Chloride in Bulk and Included in Thiourea Crystals. *Journal of Macromolecular Science, Part A: Pure and Applied Chemistry* **2009**, *46*, 16–24, doi:10.1080/10601320802511711.

83. Borisov, I.; Ovcharova, A.; Bakhtin, D.; Bazhenov, S.; Volkov, A.; Ibragimov, R.; Gallyamov, R.; Bondarenko, G.; Mozhchil, R.; Bildyukevich, A.; et al. Development of Polysulfone Hollow Fiber Porous Supports for High Flux Composite Membranes: Air Plasma and Piranha Etching. *Fibers* **2017**, *5, 6*, doi:10.3390/fib5010006.
